# Smile! (Life will be better)

**DOI:** 10.1007/s40620-020-00779-0

**Published:** 2020-06-17

**Authors:** Vittoria Esposito, Giuseppe Sileno, Silvio Abati, Marco Colucci, Massimo Torreggiani, Fabrizio Grosjean, Ciro Esposito

**Affiliations:** 1 Nephrology and Dialysis Unit, ICS Maugeri SpA SB, Pavia, Italy; 2 Dental Clinic, IRCCS San Raffaele, Segrate, Italy; 3 Nephrology and Dialysis Unit, Policlinico San Matteo, Pavia, Italy; 4 Nephrology and Dialysis Unit, University of Pavia, Via Maugeri 10, 27100 Pavia, Italy

**Keywords:** Cyclosporine, Advere events, Gingival, Kidney transplant

Z.G. 57 years-old, male was referred to our out patient clinic for follow up, 6 years after a kidney transplant. His past medical history was remarkable for polycystic kidney disease (ADPKD) and arterial hypertension treated with calcium channel blockers. In 1997 he underwent aneurysmectomy of the anterior communicating artery. In 2001 he developed dilated cardiomyopathy with 30% EF and progressive reduction of renal function. In 2003 the patient started extracorporeal dialysis with improvement of clinical and hemodynamic conditions. In 2004 he underwent left nephrectomy. In 2007 PTCA and AVI stenting and ICD placement. In 2010 kidney transplantation associated with right nephrectomy. After the surgery immediate recovery of urinary output. A triple immunosuppressive regimen with cyclosporine, mycophenolate and prednisone was started. An increase of CMV-DNA copies in the early post transplant period was treated successfully with valgancyclovir. Since 2013 substantially stable kidney function with serum creatinine around 1.6–1.7 mg/dl and absence of proteinuria. Cardiological consults performed in the post transplantation period showed stability of the cardiac conditions with preserved cardiac function. A cardiac ultrasound performed in 2016 showed left ventricular hypertrophy EF of 56% and PAPs 35 mmHg. In September 2016 the patient was referred for follow-up to our Nephrology Unit. At the first visit the patient presented with severe gingival hyperplasia (Fig. 1a, b) complaining of growing difficulties in eating and inability to perform oral hygiene. He felt miserable for his appearance that made him uneasy to relate with others. The patient reported frequent dental interventions in order to solve the problem.

**Fig. 1:**
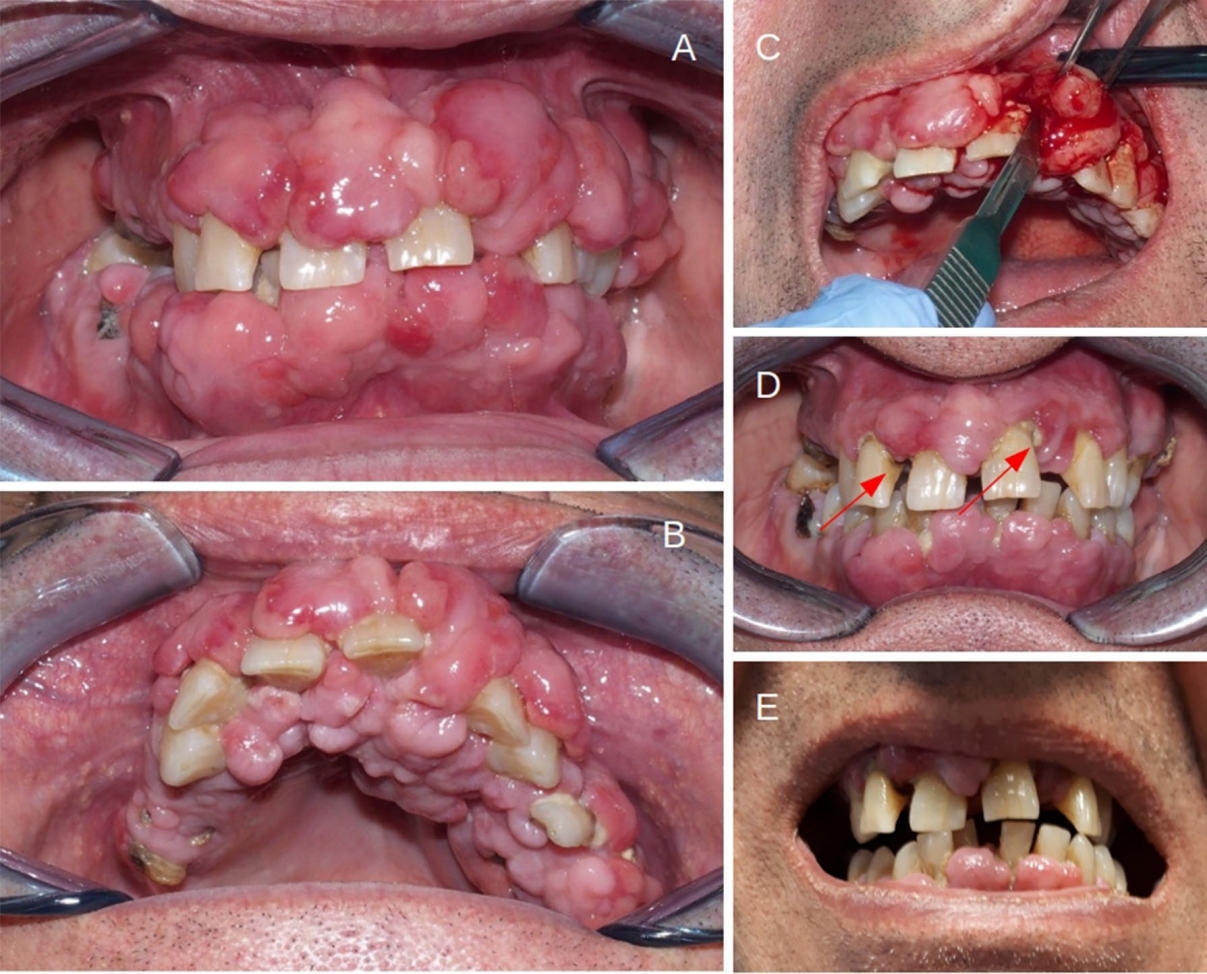
Cyclosporine-induced gingival overgrowth in a kidney transplant patient. **a** Gingival labial surface; **b** anterior palate; **c** scalpel gingivectomy; **d** dental plaques; **e** regression of gingival overgrowth after cyclosporine withdrawal

## What is gingival overgrowth and which are the main predisposing factors?

Gingival overgrowth mainly affecting the anterior teeth, more pronounced on the labial surface (Fig. 1a) but also present on the anterior palate (Fig. 1b) is an adverse effect of the systemic administration of certain drugs such as phenytoin, calcium channel blockers and cyclosporine A [1]. Its incidence was particularly elevated two decades ago when cyclosporine was the most common immunosuppressive drug prescribed to kidney transplanted recipients to prevent acute rejection, with incidence rates ranging from 13 to 84%. The effect of cyclosporine on gingival growth is amplified by the association with calcium channel blockers [2]. Age, gender, smoking habits, age at transplantation, duration of therapy and CyA dosage and poor oral hygiene represent risk factors for the development and the severity of gingival overgrowth.

## What are the mechanisms leading to cyclosporine-induced gingival overgrowth?

Gingival hypertrophy is histologically characterized by an increase in matrix deposition with proliferation of fibroblasts and inflammatory cells. It is well known that cyclosporine stimulates the deposition of matrix components [3]. At gum level it appears to promote gingival fibroblast IL-6 synthesis which increases collagen production. The susceptibility to gingival overgrowth seems to be increased by polymorphisms of MDR1 gene, encoding for P-glycoprotein. P-glycoprotein is part of ABC family of transporters and is expressed in the ducts of salivary gland having a role in excretion of certain drugs. A mutation of the MDR1 gene could reduce the excretion of cyclosporine, increasing its salivary concentration and its effects on gingival cells [4]. Poor oral hygiene with dental plaques (Fig. 1d, arrows) may contribute to gingival overgrowth triggering inflammatory changes and, through the release of mediators of inflammation, favor gingival growth.

## Gingival overgrowth management

Oral hygiene is the first of several approaches proposed for the management of gingival overgrowth [5]. Surgical treatment including scalpel gingivectomy as shown in Fig. 1c, flap surgery and laser gingivectomy should be carefully assessed since they may not be free from risks, especially infections. Furthermore relapses are not uncommon. The best therapeutic option is the withdrawal of the causative agent. Fortunately we have now alternative antirejection and antyhypertensive drugs for kidney transplant recipients. Tacrolimus causes fewer side effects than cyclosporine and as shown in our patient (Fig. 1e), it took just a few months after shifting from cyclosporine to tacrolimus and stopping nifedipine, for the gingival overgrowth to almost completely regress. Our patient’s renal function remains stable 4 years after shifting to tacrolimus.

## Conclusions

Beyond the smile, gingival overgrowth interferes with dental occlusion and speech. Furthermore it also makes oral hygiene very complicated due to frequent bleeding and pain. It makes the quality of life and relationships of the affected patients very poor. Kidney transplant is the best treatment for end stage kidney disease. With the kidney transplant program we want to give our patients an almost normal kidney function allowing them a better quality of life but we also want them to enjoy little things every day and smile.
